# Modulation of TMEM16B channel activity by the calcium-activated chloride channel regulator 4 (CLCA4) in human cells

**DOI:** 10.1016/j.jbc.2024.107432

**Published:** 2024-05-31

**Authors:** Monica Sala-Rabanal, Zeynep Yurtsever, Kayla N. Berry, Conor McClenaghan, Alyssa J. Foy, Alex Hanson, Deborah F. Steinberg, Jessica A. Greven, Colin E. Kluender, Jennifer M. Alexander-Brett, Colin G. Nichols, Tom J. Brett

**Affiliations:** 1 Center for the Investigation of Membrane Excitability Diseases (CIMED), Washington University School of Medicine, Saint Louis, Missouri, USA; 2 Department of Cell Biology and Physiology, Washington University School of Medicine, Saint Louis, Missouri, USA; 3 Division of Pulmonary and Critical Care, Department of Internal Medicine, Washington University School of Medicine, Saint Louis, Missouri, USA; 4 Immunology Program and Medical Scientist Training Program, Washington University School of Medicine, Saint Louis, Missouri, USA; 5 Department of Biochemistry and Molecular Biophysics, Washington University School of Medicine, Saint Louis, Missouri, USA

**Keywords:** calcium-activated chloride channel, calcium-activated chloride channel regulator, TMEM16 family, CLCA family, von Willebrand factor type A domain

## Abstract

The Ca^2+^-activated Cl^−^ channel regulator CLCA1 potentiates the activity of the Ca^2+^-activated Cl^−^ channel (CaCC) TMEM16A by directly engaging the channel at the cell surface, inhibiting its reinternalization and increasing Ca^2+^-dependent Cl^−^ current (I_CaCC_) density. We now present evidence of functional pairing between two other CLCA and TMEM16 protein family members, namely CLCA4 and the CaCC TMEM16B. Similar to CLCA1, (i) CLCA4 is a self-cleaving metalloprotease, and the N-terminal portion (N-CLCA4) is secreted; (ii) the von Willebrand factor type A (VWA) domain in N-CLCA4 is sufficient to potentiate I_CaCC_ in HEK293T cells; and (iii) this is mediated by the metal ion-dependent adhesion site motif within VWA. The results indicate that, despite the conserved regulatory mechanism and homology between CLCA1 and CLCA4, CLCA4-dependent I_CaCC_ are carried by TMEM16B, rather than TMEM16A. Our findings show specificity in CLCA/TMEM16 interactions and suggest broad physiological and pathophysiological links between these two protein families.

Chloride channels and their regulators underlie human physiology and pathophysiology; however, knowledge about interacting partners; and their functional consequences; is still limited. Calcium-activated chloride channel regulators (CLCAs), which have long been linked to cancer, inflammatory airway, and digestive tract diseases (1, 2), are prime examples. We first revealed the identity of a channel targeted by a CLCA protein when we demonstrated that CLCA1 binds to and potentiates the calcium-activated chloride channel (CaCC) TMEM16A (3). CLCA1 and TMEM16A have been independently linked to multiple diseases associated with anion secretion (2, 4, 5, 6, 7, 8), and CLCA1/TMEM16A interactions may explain the etiology, and perhaps serve as a therapeutic target, for some of these conditions. Both CLCA1 and TMEM16A have been linked to aging-related kidney injury, wherein CLCA1 is overexpressed leading to increased TMEM16A activity and injury-related signaling that is abrogated by TMEM16A inhibitors (9). In addition, both CLCA1 and TMEM16A have been separately implicated in colorectal (10, 11), pancreatic (12, 13), and ovarian (14, 15) cancers. Finally, both CLCA1 and TMEM16A have been suggested as therapeutic targets for cystic fibrosis; to restore chloride transport and mucus properties (8, 16, 17). Thus, the functional link between CLCA1 and TMEM16A underlies both disease pathogenesis and potential synergistic routes to treatment.

With this in mind, we sought to investigate the unexplored potential for functional linkages between other members of the CLCA and TMEM16 families. Here, we demonstrate another distinct, functional interaction between a CLCA family member and a CaCC, namely CLCA4 and TMEM16B.

## Results

### CLCA4 is a self-cleaving, membrane-anchored protein

**Figure 1:**
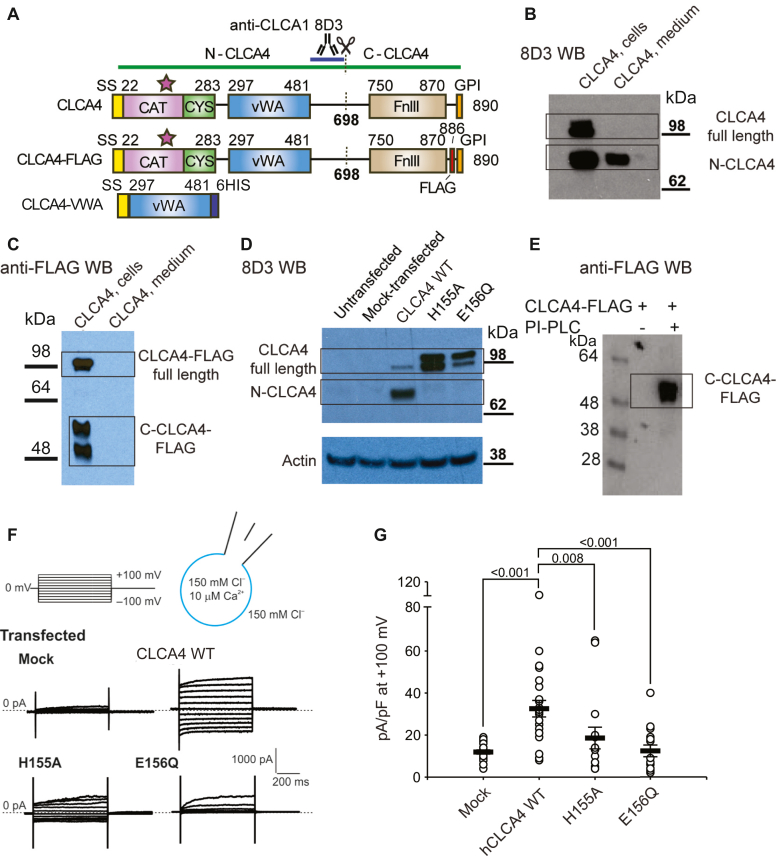
**CLCA4 self-cleavage is required to potentiate I**_**CaCC**_**in HEK293T cells.***A*, domain schematic representation of human CLCA4 constructs used in this study. The anti-CLCA1 antibody 8D3 is cross-reactive with CLCA4 and the region containing the epitope is highlighted. Domain abbreviations are as follows: CAT = MMP-like catalytic domain; SS = signal sequence; CYS = MMP-like cysteine-rich domain; VWA = von-Willebrand Type A domain; FnIII = fibronectin type-III domain; GPI = glycosylphosphatidylinositol anchor. *B–D*, CLCA4 is cleaved and secreted. *B*, Western blot (using 8D3) of cells transfected with CLCA4. Full-length CLCA4 is only detected in solubilized cells, whereas the cleaved N-terminal fragment (N-CLCA4) is found in both cell lysate and secreted into the medium. *C*, Western blot (using anti-FLAG M2) of cells transfected with CLCA4-FLAG. Both full-length and C-CLCA4 are only detected in solubilized cells. *D*, Western blot (using 8D3) of cells transfected with either WT CLCA4 or CLCA4 containing mutations to predicted active-site residues in CLCA4 MMP-like domain. These mutations (H155A and E156Q) prevent self-cleavage. *E*, anti-FLAG Western blot of cells transfected with CLCA-4-FLAG treated with PI-PLC to release GPI-anchored proteins. C-CLCA4-FLAG is released into media following PI-PLC treatment. *F*, CLCA4 self-cleavage is required to potentiate CaCC-like currents, *i.e.*, I_CaCC_. Cells were transfected with expression constructs or empty pHLsec plasmid (mock) and assayed by whole-cell patch clamp 48 h later. Representative whole-cell currents; pulse protocol on top. *G*, current density at +100 mV, measured at the end of the 600-ms voltage pulse. Symbols are data from individual patches (*n* = 10–25); bars are means ± S.E.M. of all experiments. Statistical differences between CLCA4 transfected and other conditions are indicated (Kruskal-Wallis one-way ANOVA on ranks, *H* (3) = 28.813, *p* < 0.001, followed by Dunn’s test).

CLCA proteins contain a matrix metalloprotease-like (MMP-like) domain in their N-terminus (Fig. 1*A*) (18), and this domain is responsible for CLCA1 self-cleavage (19). Since all mammalian CLCA proteins contain the catalytically required HE*XX*H*XXX*G*XX*DE motif (19), we expressed CLCA4 constructs in HEK293T cells to investigate the potential for CLCA4 self-cleavage by the MMP-like domain within this protein. CLCA4 is predicted to contain a C-terminal glycosylphosphoinositide (GPI) anchor (Fig. 1*A*) (1). Consistent with this, we identified only full-length CLCA4 in pelleted cells, while the cleaved N-terminal fragment (N-CLCA4) was released into the medium (Fig. 1*B*). A CLCA4 construct containing a FLAG epitope near the predicted GPI anchor site (CLCA4- FLAG; Fig. 1*A*) confirmed that the C-terminal fragment (C-CLCA4-FLAG) remains attached to cells: C-CLCA4-FLAG was observed only in cell pellets and was not detected in media supernatants (Fig. 1*C*). To address whether the MMP-like domain is responsible for self-cleavage, we designed mutations to catalytically required residues (H155A, E156Q). Either mutation prevented cleavage of CLCA4, producing only full-length protein (Fig. 1*D*). In order to experimentally validate the presence of the computationally predicted GPI-anchor, we treated cells expressing CLCA4-FLAG with phosphoinositol-phospholipase C (PI-PLC); this treatment resulted in the release of C-CLCA4-FLAG into the medium (Fig. 1*E*), as detected by anti-FLAG Western blot, demonstrating that CLCA4 contains a GPI-anchor. Thus, the self-cleavage of CLCA4 releases N-CLCA4, leaving C-CLCA4 attached to the cell membrane through a GPI anchor.

### CLCA4 self-cleavage activates Cl channels

Several CLCA family members have been shown to stimulate calcium-activated chloride currents (I_CaCC_) in cells in overexpression studies (2, 20); however, this has not yet been demonstrated for CLCA4. Whole-cell patch clamp experiments on HEK293T cells transfected with WT CLCA4 demonstrated robust currents resembling those mediated by Ca^2+^ - activated chloride channels (I_CaCC_), while such currents were absent from mock-transfected cells (Fig. 1, *F* and *G*). We previously showed that self-cleavage regulates the ability of CLCA1 to potentiate TMEM16A (19). Here, we sought to address whether self-cleavage of CLCA4 may serve a similar role, by comparing the electrogenic properties of wild-type (WT) CLCA4 with those of the cleavage-deficient mutants H155A and E156Q (Fig. 1, *F* and *G*). I_CaCC_ were absent in cells transfected with catalytically deficient CLCA4 mutants (Fig. 1, *F* and *G*), indicating that self-cleavage of CLCA4 is required to release N-CLCA4 into the extracellular milieu and potentiate I_CaCC_.

### CLCA4 potentiates TMEM16B

**Figure 2:**
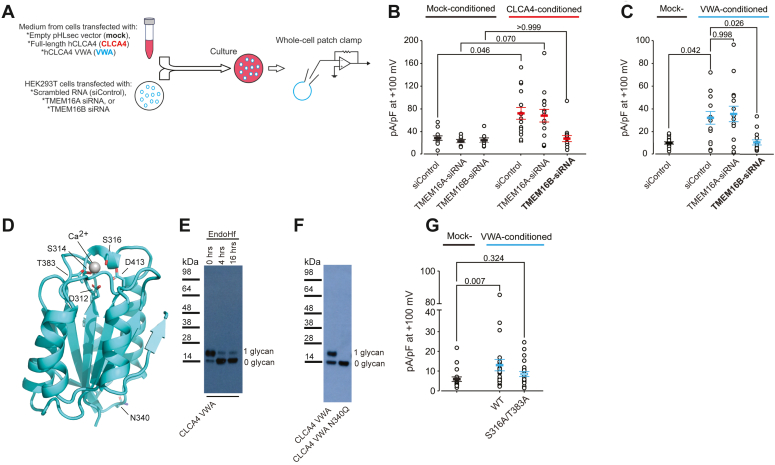
**CLCA4-dependent I**_**CaCC**_**are carried by TMEM16B, and paracrine CLCA4-TMEM16B interactions are mediated by a MIDAS motif in the CLCA4 VWA domain.***A*, HEK293T cells transfected with scramble (siControl), TMEM16A siRNA, or TMEM16B siRNA were cultured in medium from mock-transfected cells or from cells expressing full-length CLCA4 or CLCA4 VWA domain (VWA), and assayed by whole-cell patch clamp. *B* and *C*, I_CaCC_ were activated in CLCA4- (*B*) and VWA-conditioned (*C*) cells transfected with siControl and TMEM16A siRNA, but current density remained at background levels in cells transfected with TMEM16B siRNA. *D*, homology model of the CLCA4 VWA domain, based on the crystal structure of CLCA1 VWA (PDB ID: 6PYO) (22). Residues composing the MIDAS site and the N-linked glycosylation site (N340) are shown as sticks. *E*, purified CLCA4 VWA expressed in the presence of kifunensine was treated with the deglycosylation enzyme EndoHf, resulting in a shift to lower molecular weight. *F*, mutation of the CLCA4 VWA glycosylation site (N340Q) results in a single band corresponding to the non-glycosylated protein. *G*, CLCA4-dependent I_CaCC_ potentiation was reduced in naïve HEK293T cells conditioned with MIDAS motif double mutant S316A/T383A VWA. Experiments were performed as in (*A*). *B*, *C* and *G*, current density at +100 mV, measured at the end of the 600-ms voltage pulse (protocol as in Fig. 1*F*). Symbols are data from individual patches (*n* = 10–25); bars are means ± S.E.M. of all experiments. Statistical differences were evaluated by Kruskal-Wallis one-way ANOVA on ranks, as follows: (*B*) *H* (5) = 26.647, *p* < 0.001; (*C*) *H* (3) = 15.780, *p* = 0.001; and (*G*) *H* (2) = 9.267, *p* = 0.010. *Post hoc* all-pairwise comparisons between groups were determined by Dunn’s method. Statistical differences are indicated in (*B*) between mock- and CLCA4-conditioned media for each siRNA transfection in CLCA4-conditioned *versus* scrambled siRNA control in mock-conditioned. Statistical differences are indicated in (*C*) between mock- and CLCA4-conditioned media for each siRNA transfection. Statistical differences are indicated in (*G*) between all treatments.

We previously showed that CLCA1 potentiates the CaCC TMEM16A in a paracrine manner (3). To investigate whether the CLCA4induces CaCC *via* a similar mechanism, we transfected HEK293T cells with either siRNA targeting TMEM16A or scrambled siRNA control, and then cultured them in media conditioned by mock-transfected control cells or cells transfected with full-length CLCA4 (Fig. 2*A*). Control (siRNA-transfected) cells in CLCA4-conditioned media, but not cells in mock-conditioned media, displayed robust I_CaCC_. Similar currents were activated in CLCA4-conditioned cells transfected with TMEM16A siRNA, which is not consistent with CLCA4 acting on TMEM16A (Fig. 2*B*). Since TMEM16B displays sequence and functional similarity to TMEM16A and is also expressed in HEK293T cells (21), we hypothesized that these currents activated by CLCA4 might instead be carried by TMEM16B. Consistent with this hypothesis, currents in CLCA4-conditioned cells transfected with TMEM16B siRNA were not significantly different than mock-conditioned cells (Fig. 2*B*). Together, our results are consistent with N-CLCA4 potentiating TMEM16B currents in HEK293T cells *via* a paracrine mechanism.

### CLCA4 VWA MIDAS motif mediates TMEM16B interaction and potentiation

We previously demonstrated that the CLCA1 von Willebrand type A (VWA) domain is necessary and sufficient to potentiate currents through TMEM16A, and that this interaction is dependent on the VWA metal-ion-dependent adhesion site (MIDAS) motif (17). To investigate whether CLCA4-TMEM16B interactions are mediated similarly, we carried out experiments with only the CLCA4 VWA domain (CLCA4 VWA) (Fig. 2, *C–F*). We built a CLCA4 VWA homology model (Fig. 2*D*) based on our high-resolution crystal structure of CLCA1 VWA (22), and identified a putative MIDAS motif. Purified CLCA4 VWA migrated as a doublet on SDS-PAGE (Fig. 2*E*); due to an N-linked glycosylation site (N340) that is predicted to be located distal to the predicted MIDAS motif. The glycosylation site was verified by treatment of CLCA4 VWA with the deglycosylating enzyme EndoHf and by mutation (N340Q), both of which resulted in a shift to a single lower molecular weight species (Fig. 2*F*). As observed with N-CLCA4, CLCA4 VWA markedly potentiated I_CaCC_ in HEK293T cells transfected with scrambled siRNA, and similarly potentiated currents were seen in cells transfected with TMEM16A siRNA, but not with TMEM16B siRNA (Fig. 2*C*). While there was a significant potentiation of I_CaCC_ by media conditioned by isolated WT VWA domain (Fig. 2*G*), there was no significant potentiation by media conditioned by isolated VWA domain containing mutations S316A and T383A (Fig. 2*G*) in the predicted CLCA4 MIDAS motif that are equivalent to those critical for CLCA1 VWA-TMEM16A interactions (17) (Fig. 2*D*). In summary, in parallel to the activation of TMEM16A, the above results show that CLCA4 VWA modulates the activity of TMEM16B in a MIDAS motif-dependent manner.

## Discussion

### CLCA family of TMEM16 regulators

**Figure 3:**
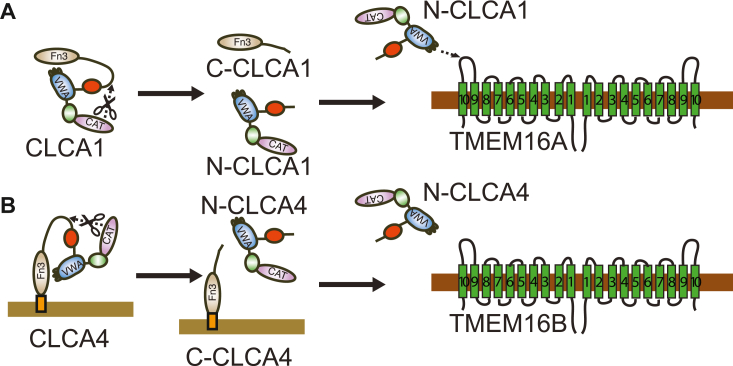
**Working model for CLCA family self-activation and engagement of TMEM16 family proteins.***A*, soluble CLCA1 undergoes self-cleavage mediated by its MMP-like domain (CAT) to produce the N-CLCA1 and C-CLCA1 fragments. N-CLCA1 engages TMEM16A α9-α10 loop using VWA MIDAS motif. *B*, membrane-anchored CLCA4 undergoes self-cleavage mediated by its MMP-like domain (CAT) to produce the N-CLCA4 and C-CLCA4 fragments. N-CLCA4 engages TMEM16B using its VWA domain.

TMEM16A and TMEM16B generate Ca^2+^-activated chloride channels in multiple cell types, and regulate multiple physiological systems, including exocrine and endocrine secretion, fertilization, olfactory sensation, and smooth muscle contractility (23, 24). They are activated by internal Ca^2+^ directly and through interaction with calmodulin and calmodulin-dependent protein kinase II (CaMKII). As we have shown, TMEM16A is also stabilized at the cell membrane by interaction with secreted CLCA1 (3, 17). Here, we demonstrate that an additional CLCA family member, CLCA4 also activates TMEM16B channels, by a parallel mechanism. Our working model (Fig. 3) is that both CLCA1 and CLCA4 include a signal sequence and are produced in the secretory pathway. Each also contains an N-terminal MMP-like domain, which cleaves the protein into N- and C-terminal fragments. Whereas N-CLCA1 and C-CLCA1 are both soluble (Fig. 3*A*), cleavage of CLCA4 releases N-CLCA4 but leaves C-CLCA4 attached to the plasma membrane *via* a GPI anchor (Fig. 3*B*). The VWA domains of N-CLCA1 and N-CLCA4 engage their respective target channels, namely TMEM16A and TMEM16B, *via* a MIDAS motif-mediated interaction. Because of their specificity, CLCA1 VWA and CLCA4 VWA may serve as reagents to identify, differentiate, and characterize TMEM16A and TMEM16B in biochemical, cellular, and physiological systems.

### A CLCA4/TMEM16B cooperative sphere?

The CLCA4-mediated potentiation of TMEM16B unveiled here hints at cooperative roles for this pair in health and disease, and CLCA4 and TMEM16B also appear to overlap at the expression, functional, and pathophysiological levels. CLCA4 is found in tissues with vast mucosal epithelial surfaces, including the lungs (25, 26, 27), intestine (26, 28), and bladder (29) as well as in the brain (26); TMEM16B shows an overlapping expression pattern: in lung, esophagus, and nasal passage mucosae (30), intestines, brain (31, 32), and eye (33). TMEM16B has been linked to transepithelial fluid transport, smooth muscle contraction, and neuronal excitability (34), and reduced CLCA4 expression has been observed in diseases hallmarked by disrupted transepithelial secretion, such as cystic fibrosis (35) and Crohn’s disease (28). TMEM16B and CLCA4 have also both been linked to cancer (29, 36, 37, 38).

Finally, our results not only pave the way for a deeper understanding of the physiological roles of CLCA4 and TMEM16B, but the apparent specificity of CLCA1/TMEM16A and CLCA4/TMEM16B pairing further hints at the possibility of additional specific pairings between members of each family. CLCA2 activates ion currents, and while CLCA2 overexpression studies suggest that it may be TMEM16A(21), the channel responsible has yet to be identified.

## Experimental procedures

### Expression constructs

We created a full-length untagged human CLCA4 (22–917) by cloning it into pHLsec (Addgene, Watertown, MA). We created a FLAG-tagged full-length human CLCA4 (22–917) by inserting a FLAG sequence (DYKDDDDK) between the predicted FnIII and GPI anchor domains (CLCA4-FLAG) (Fig. 1*A*). We created a CLCA4 VWA (297–481) by cloning into the pHLsec vector, which includes a C-terminal hexahistidine tag (39). Domain boundaries were predicted by means of the Phyre2 Protein Fold Recognition Server (40). Mutations in the metalloprotease domain of full-length CLCA4, *i.e.* H155A and E156Q, and in the metal ion-dependent adhesion site (MIDAS) motif of the VWA domain, *i.e.* T383A and S316A were introduced by means of the Q5 Site-Directed Mutagenesis Kit (New England Biolabs) and verified by sequencing. HEK293T cells were cultured and transfected as described (3), and expression and secretion of CLCA4 proteins were confirmed by Western blot.

### Western blot

General methods were as described (3). Mouse anti-human CLCA1 antibody 8D3 developed in-house (1:1000; (25)) and goat anti-mouse IgG antibody-HRP conjugate (1:10,000; Santa Cruz Biotechnology) were used to detect N-terminal CLCA4; and monoclonal anti-FLAG M2-Peroxidase (HRP) antibody (1:5000; MilliporeSigma, Saint Louis, MO) was used to detect C-terminal CLCA4. Rabbit anti-6-His-antibody-HRP conjugate (1:10,000; Bethyl Laboratories) was used to detect VWA proteins. For cell lysate blots, mouse anti-actin monoclonal antibody C4 (1:5000; MilliporeSigma) and goat anti-mouse IgG-HRP conjugate (as above) were used as a protein loading control. Analysis of WT and mutant CLCA4 constructs by cellular expression and Western blot were carried out in triplicate. Results were consistent between replicates, with representative results shown. The anti-CLCA1 8D3 antibody was validated as being cross-reactive with CLCA4 by using a CLCA4 construct containing an N-term myc tag that we published previously (19). Briefly, the construct was expressed in HEK293 cells, samples of supernatant and cells were collected, run on SDS-PAGE, and analyzed by Western blot. N-CLCA4 was detected on Western blot by both the anti-myc antibody and 8D3, therefore validating that the anti-CLCA1 antibody 8D3 was crossreactive with CLCA4.

### siRNA Knockdown of TMEM16A and TMEM16B

Silencing of TMEM16A or TMEM16B was performed as reported (3). TMEM16A siRNA (5′-AAG UUA GUG AGG UAG GCU GGG AAC C-3′; HSS123904, Life Technologies), TMEM16B siRNA (5′-GGG AGG AAT TTG AGC ACA ATC TGA T-3′; HSS125973, Life Technologies) or medium GC-content Stealth RNAi negative control (5′-GGU UCC CAG CCU ACC UCA CUA ACU U-3′; 12935300, Life Technologies) were used.

### PI-PLC Digestion of GPI-anchored C-CLCA4

HEK293T cells were grown to 50% confluence and transfected with CLCA4-FLAG. Twenty-four hours after transfection, cells were chilled on ice, rinsed twice with chilled phosphate-buffered saline (PBS), and then exposed to 0.5 ml PBS in the absence (control) or presence of 0.1 units of phosphatidylinositol-specific phospholipase C (PI-PLC). The plate was rocked for 20 min at 4 °C, and 1 ml samples of supernatant were collected from each well, concentrated (Amicon centrifugal microconcentrator), separated, and analyzed by SDS-PAGE and anti-FLAG Western blot. Cellular assay and Western blot analysis were carried out in duplicate. Results were consistent, with representative results shown.

### Whole-cell patch clamp recordings

Transfected or conditioned HEK293T cells were tested for I_CaCC_; general procedures, data acquisition, and analysis were as described (3). Pipette (∼3 MΩ) solution contained 142 mM N-methyl-D-glucamine chloride, 10 mM Hepes, 2 mM MgCl_2_, 5 mM EGTA, and 4.9 mM CaCl_2_ to attain 10 μM free Ca^2+^ at pH 7.1, per MaxChelator (41). The extracellular solution was 150 mM NaCl, 10 mM Hepes, 1 mM CaCl_2,_ and 1 mM MgCl_2_, at pH 7.4. Results are mean ± S.E.M. Statistical differences were evaluated by Kruskal-Wallis one-way ANOVA on ranks and Dunn’s multiple comparisons *post hoc* test (SigmaPlot 14.5; Systat Software).

## Data availability

All data described in this study are contained within the main article.

## Conflict of interest

The authors declare that they have no known competing financial interests or personal relationships that could have appeared to influence the work reported in this paper.

## References

[1] Patel A.C., Brett T.J., Holtzman M.J. (2009). The role of CLCA proteins in inflammatory airway disease. Annu. Rev. Physiol..

[2] Sala-Rabanal M., Yurtsever Z., Berry K.N., Brett T.J. (2015). Novel roles for chloride channels, exchangers, and regulators in chronic inflammatory airway diseases. Mediators Inflamm..

[3] Sala-Rabanal M., Yurtsever Z., Nichols C.G., Brett T.J. (2015). Secreted CLCA1 modulates TMEM16A to activate Ca(2+)-dependent chloride currents in human cells. Elife.

[4] Liu C.L., Shi G.P. (2019). Calcium-activated chloride channel regulator 1 (CLCA1): more than a regulator of chloride transport and mucus production. World Allergy Organ J..

[5] Thiagarajah J.R., Donowitz M., Verkman A.S. (2015). Secretory diarrhoea: mechanisms and emerging therapies. Nat. Rev. Gastroenterol. Hepatol..

[6] van der Doef H.P., Slieker M.G., Staab D., Alizadeh B.Z., Seia M., Colombo C. (2010). Association of the CLCA1 p.S357N variant with meconium ileus in European patients with cystic fibrosis. J. Pediatr. Gastroenterol. Nutr..

[7] Galietta L.J.V. (2022). TMEM16A (ANO1) as a therapeutic target in cystic fibrosis. Curr. Opin. Pharmacol..

[8] Danahay H.L., Lilley S., Fox R., Charlton H., Sabater J., Button B. (2020). TMEM16A potentiation: a novel therapeutic approach for the treatment of cystic fibrosis. Am. J. Respir. Crit. Care Med..

[9] Lee H.J., Donati A., Feliers D., Sun Y., Ding Y., Madesh M. (2021). Chloride channel accessory 1 integrates chloride channel activity and mTORC1 in aging-related kidney injury. Aging Cell.

[10] Yan Y., Ding X., Han C., Gao J., Liu Z., Liu Y. (2022). Involvement of TMEM16A/ANO1 upregulation in the oncogenesis of colorectal cancer. Biochim. Biophys. Acta Mol. Basis Dis..

[11] Wei F.Z., Mei S.W., Wang Z.J., Chen J.N., Shen H.Y., Zhao F.Q. (2020). Differential expression analysis revealing CLCA1 to Be a prognostic and diagnostic biomarker for colorectal cancer. Front. Oncol..

[12] Hu D., Ansari D., Zhou Q., Sasor A., Hilmersson K.S., Bauden M. (2018). Calcium-activated chloride channel regulator 1 as a prognostic biomarker in pancreatic ductal adenocarcinoma. BMC Cancer.

[13] Crottes D., Lin Y.T., Peters C.J., Gilchrist J.M., Wiita A.P., Jan Y.N. (2019). TMEM16A controls EGF-induced calcium signaling implicated in pancreatic cancer prognosis. Proc. Natl. Acad. Sci. U. S. A..

[14] Liu Z., Zhang S., Hou F., Zhang C., Gao J., Wang K. (2019). Inhibition of Ca(2+) -activated chloride channel ANO1 suppresses ovarian cancer through inactivating PI3K/Akt signaling. Int. J. Cancer.

[15] Musrap N., Tuccitto A., Karagiannis G.S., Saraon P., Batruch I., Diamandis E.P. (2015). Comparative proteomics of ovarian cancer aggregate formation reveals an increased expression of calcium-activated Chloride Channel Regulator 1 (CLCA1). J. Biol. Chem..

[16] Centeio R., Ousingsawat J., Schreiber R., Kunzelmann K. (2021). CLCA1 regulates airway mucus production and ion secretion through TMEM16A. Int. J. Mol. Sci..

[17] Sala-Rabanal M., Yurtsever Z., Berry K.N., Nichols C.G., Brett T.J. (2017). Modulation of TMEM16A channel activity by the von Willebrand factor type A (VWA) domain of the calcium-activated chloride channel regulator 1 (CLCA1). J. Biol. Chem..

[18] Lenart A., Dudkiewicz M., Grynberg M., Pawlowski K. (2013). CLCAs - a family of metalloproteases of intriguing phylogenetic distribution and with cases of substituted catalytic sites. PLoS One.

[19] Yurtsever Z., Sala-Rabanal M., Randolph D.T., Scheaffer S.M., Roswit W.T., Alevy Y.G. (2012). Self-cleavage of human CLCA1 protein by a novel internal metalloprotease domain controls calcium-activated chloride channel activation. J. Biol. Chem..

[20] Loewen M.E., Forsyth G.W. (2005). Structure and function of CLCA proteins. Physiol. Rev..

[21] Sharma A., Ramena G., Yin Y., Premkumar L., Elble R.C. (2018). CLCA2 is a positive regulator of store-operated calcium entry and TMEM16A. PLoS One.

[22] Berry K.N., Brett T.J. (2020). Structural and biophysical analysis of the CLCA1 VWA domain suggests mode of TMEM16A engagement. Cell Rep..

[23] Agostinelli E., Tammaro P. (2022). Polymodal control of TMEM16x channels and scramblases. Int. J. Mol. Sci..

[24] Hawn M.B., Akin E., Hartzell H.C., Greenwood I.A., Leblanc N. (2021). Molecular mechanisms of activation and regulation of ANO1-Encoded Ca(2+)-Activated Cl(-) channels. Channels (Austin).

[25] Alevy Y.G., Patel A.C., Romero A.G., Patel D.A., Tucker J., Roswit W.T. (2012). IL-13-induced airway mucus production is attenuated by MAPK13 inhibition. J. Clin. Invest..

[26] Piirsoo M., Meijer D., Timmusk T. (2009). Expression analysis of the CLCA gene family in mouse and human with emphasis on the nervous system. BMC Dev. Biol..

[27] Plog S., Grotzsch T., Klymiuk N., Kobalz U., Gruber A.D., Mundhenk L. (2012). The porcine chloride channel calcium-activated family member pCLCA4a mirrors lung expression of the human hCLCA4. J. Histochem. Cytochem..

[28] Comelli E.M., Lariani S., Zwahlen M.C., Fotopoulos G., Holzwarth J.A., Cherbut C. (2009). Biomarkers of human gastrointestinal tract regions. Mamm. Genome.

[29] Hou T., Zhou L., Wang L., Kazobinka G., Zhang X., Chen Z. (2017). CLCA4 inhibits bladder cancer cell proliferation, migration, and invasion by suppressing the PI3K/AKT pathway. Oncotarget.

[30] Uhlen M., Fagerberg L., Hallstrom B.M., Lindskog C., Oksvold P., Mardinoglu A. (2015). Proteomics. Tissue-based map of the human proteome. Science.

[31] Li K.X., He M., Ye W., Simms J., Gill M., Xiang X. (2019). TMEM16B regulates anxiety-related behavior and GABAergic neuronal signaling in the central lateral amygdala. Elife.

[32] Wang L., Simms J., Peters C.J., Tynan-La Fontaine M., Li K., Gill T.M. (2019). TMEM16B calcium-activated chloride channels regulate action potential firing in lateral septum and aggression in male mice. J. Neurosci..

[33] Keckeis S., Reichhart N., Roubeix C., Strauss O. (2017). Anoctamin2 (TMEM16B) forms the Ca(2+)-activated Cl(-) channel in the retinal pigment epithelium. Exp. Eye Res..

[34] Le S.C., Yang H. (2021). Structure-Function of TMEM16 ion channels and lipid scramblases. Adv. Exp. Med. Biol..

[35] Ritzka M., Stanke F., Jansen S., Gruber A.D., Pusch L., Woelfl S. (2004). The CLCA gene locus as a modulator of the gastrointestinal basic defect in cystic fibrosis. Hum. Genet..

[36] Pedemonte N., Galietta L.J. (2014). Structure and function of TMEM16 proteins (anoctamins). Physiol. Rev..

[37] Wei L., Chen W., Zhao J., Fang Y., Lin J. (2020). Downregulation of CLCA4 expression is associated with the development and progression of colorectal cancer. Oncol. Lett..

[38] Yu Y., Walia V., Elble R.C. (2013). Loss of CLCA4 promotes epithelial-to-mesenchymal transition in breast cancer cells. PLoS One.

[39] Aricescu A.R., Lu W., Jones E.Y. (2006). A time- and cost-efficient system for high-level protein production in mammalian cells. Acta Crystallogr. D Biol. Crystallogr..

[40] Kelley L.A., Mezulis S., Yates C.M., Wass M.N., Sternberg M.J. (2015). The Phyre2 web portal for protein modeling, prediction and analysis. Nat. Protoc..

[41] Bers D.M., Patton C.W., Nuccitelli R. (1994). A practical guide to the preparation of Ca2+ buffers. Methods Cell Biol..

